# A Population-Based Study of Peyronie's Disease: Prevalence and Treatment Patterns in the United States

**DOI:** 10.1155/2011/282503

**Published:** 2011-10-23

**Authors:** Dana Britt DiBenedetti, Dat Nguyen, Laurie Zografos, Ryan Ziemiecki, Xiaolei Zhou

**Affiliations:** ^1^RTI Health Solutions, RTI International, Research Triangle Park, NC 27709, USA; ^2^Auxilium Pharmaceuticals, Inc., Malvern, PA 19355, USA

## Abstract

*Purpose*. To estimate the US prevalence of Peyronie's disease (PD) from patient-reported data and to identify diagnosis and treatment patterns. *Methods*. 11,420 US males ≥18 years old completed a brief web-based survey regarding the presence of PD, past treatments, and penile symptoms (Phase 1). Phase 1 respondents with PD diagnosis, history of treatment, or PD-related symptoms then completed a disease-specific survey (Phase 2). *Results*. Estimated prevalence of PD ranged from 0.5% (diagnosis of PD) to 13% (diagnosis, treatment, or penile symptoms). Thirty-six percent of Phase 2 participants reported that penile symptoms interfered with sexual activities. Of participants who sought treatment for penile symptoms (*n* = 128), 73% initially saw a primary care physician, 74% did not receive treatment from their first doctor, and 92% were not diagnosed with PD. *Conclusions*. PD may be underdiagnosed/undertreated in the US. Improved awareness is needed of PD symptoms and treatment options among health care professionals.

## 1. Introduction

Peyronie's disease (PD) is a progressive fibrotic tissue disorder with unknown etiology [1–3]. It is a connective tissue disorder of the penile tunica albuginea that results in the formation of a palpable scar or hard plaque, most commonly on the dorsal surface of the penis, which may cause a curvature deformity and changes in the length or circumference of the penis while erect. Penile folding or collapsing during intercourse, penile pain, and erectile dysfunction (ED) are also associated with PD [1]. In a recent retrospective study of 1,001 patients with PD, 58.1% of patients reported having ED [4]. PD may also limit the ability to have intercourse and may make intercourse less enjoyable, more awkward, and even impossible. PD can be psychologically and physically devastating for patients and can also negatively impact partner relationships [5, 6]. 

The disease is not well understood, and consequently, patients are often misdiagnosed (e.g., with ED) and the time to diagnosis and treatment can be long. Current theories regarding the cause of PD suggest that a variety of factors may be involved, such as a traumatic event, sometimes related to sexual intercourse, the presence of genetic predisposition, or occurrence of abnormal wound healing, including involvement of transforming growth factor *β*-1 or other profibrotic factors [7, 8]. Convincing evidence regarding the pathophysiology, diagnosis, and treatment of PD continues to elude physicians [9].

To date, there are no Food and Drug Administration- (FDA)-approved, nonsurgical options for treatment of PD, and clinically proven treatment options are limited. Current nonsurgical treatment options for PD include oral, transdermal, or intralesional injection, extracorporeal shock wave therapy, or external traction therapy [1, 10–13]. The efficacy of many nonsurgical treatments is unknown due to underpowered and methodologically heterogeneous treatment studies [10]. Surgical interventions for PD may follow unsuccessful nonsurgical treatment options and are primarily considered for patients with deformity that impairs sexual function [14]. It is recommended that surgery be delayed until the disease has stabilized for at least 6 to 12 months and the active inflammatory disease process involving penile pain and recent changes in penile deformity has concluded [1]. Surgical correction has its limitations, including penile shortening in many procedures, recurrence of curvature, altered penile sensation, development of new-onset ED, and the potential rare complications of surgery, such as bleeding or infection [14–16].

The prevalence of PD is not known. Studies of PD prevalence are limited and inconsistent; estimates have ranged from 0.39% to greater than 20% (see Table 1) [17–24]. Prevalence can range based on study design or inclusion of patients with different comorbidities within the study population (e.g., older age, diabetes, and ED). In addition, the actual occurrence of this disease within the population may be higher due to patients' reluctance to come to their physician for treatment and diagnosis of this embarrassing condition [25]. Limited understanding of PD in the medical community may also contribute to underdiagnosis. 

The current study was motivated by the lack of the current PD epidemiological data from the general US population and the need to understand how patients and physicians perceive this disease in regards to diagnosis and treatment. The current study is web-based survey of US male adults aged 18 years or older who were enrolled in a probability-based panel of research subjects representative of the full US population (Knowledge Networks; KN) [26]. The study objectives were (1) to estimate the prevalence of PD in the general US male population, (2) to determine the time from first symptom noticed to medical treatment, and (3) to describe diagnosis and treatment patterns. Analyses of these objectives were based on patient-reported data.

## 2. Methods

### 2.1. Study Design

**Figure 1:**
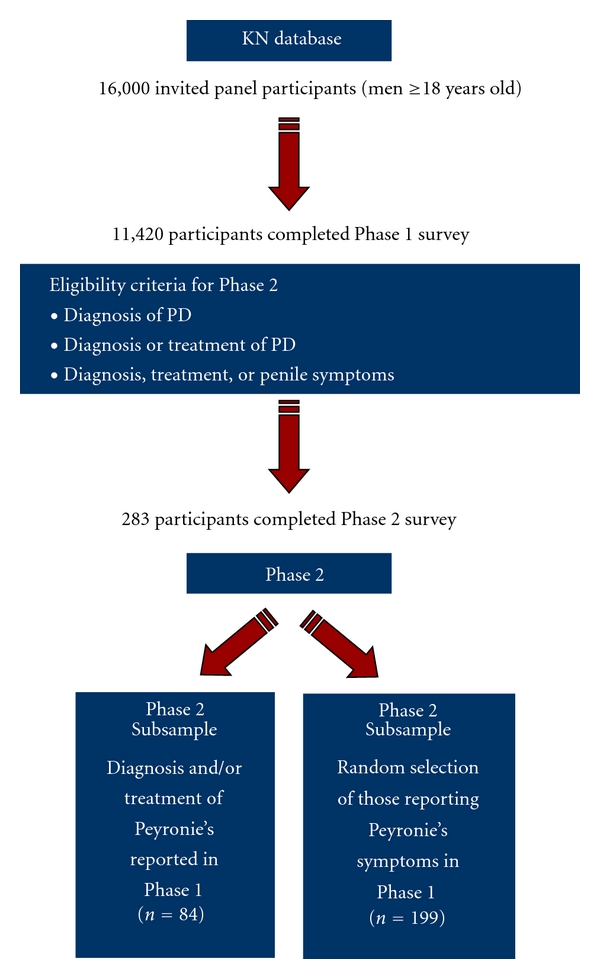
Diagram of study design. Data for this study were collected in 2 phases. In Phase  1, panel participants were screened for the presence of symptoms, past treatment, and a diagnosis of Peyronie's disease. In Phase  2, eligibility to complete the full survey was determined by participants' responses to the screening items in Phase  1. KN = Knowledge Networks.

This cross-sectional, population-based survey was conducted in 2 phases (Figure 1). In the initial phase (Phase  1), participants from the KN online panel were screened for the presence of current symptoms of PD, past surgical or nonsurgical treatments, and a diagnosis of PD. Data from Phase  1 was used to estimate the prevalence of PD in the general US population and to determine eligibility to complete the full survey in Phase  2. In the Phase  2 portion of the study, a subsample of Phase  1 participants meeting the eligibility criteria for PD were invited to complete the PD survey (see the Appendix), which focused on the presence and severity of current symptoms, family history, and treatment history for penile symptoms. 

The KN panel, a proprietary web-enabled panel of individuals who have agreed to participate in ongoing survey research is the only known online panel based on a random-digit-dialing sample of the full US population, ensuring that the panel is representative of the US population [26]. KN provides panelists “points” that can be redeemed for cash at regular intervals for panel participation (for those with Internet access) or with web-enabled technology to ensure that those who would not otherwise have access to the Internet are able to participate in KN surveys [26]. The KN panel has been used to estimate prevalence previously in several different studies [27–29]. 

The current study was structured in a way that allowed for patient confidentiality in both phases: identifying patient information was never transmitted or provided to study personnel and KN did not link patient contact information and survey responses. Following ethics committee approval by RTI International's Institutional Review Board, screening in Phase  1 was fielded from November 6, 2007, through December 9, 2007, and the Phase 2 survey was fielded from March 20, 2008, through March 31, 2008. Prior to administration of either Phase 1 or Phase 2 surveys, all participants provided written informed consent.

### 2.2. Participants

A total of 16,000 men aged 18 years and older residing in the US were randomly selected from the KN online panel and invited to participate in the current study. Individuals eligible for Phase 1 included all adult men (aged 18 years or older) who were KN panel members at the time of Phase 1 recruitment. Eligibility for the PD full survey (Phase 2) was based on responses to the screening questions in Phase 1. Specifically, Phase 1 respondents eligible for Phase 2 were those who reported 1 or more of the following criteria at screening:

1. ever received a diagnosis of PD by a doctor or other health care professional,
2. ever received either surgery or a nonsurgical procedure to correct the shape of the penis,
3. currently have at least 1 of the following symptoms:
  - a lump or bump (excluding genital warts, pimples, and blisters) under the skin of the penis when the penis is not erect,
  - an area of unusual firmness or hardened tissue (excluding genital warts, pimples, and blisters) under the skin of the penis when the penis is not erect,
  - a change in erect penis that includes at least 1 of the following symptoms:
  - a significant bend or curve of the penis,
  - an indentation on 1 or both sides of the penis,
  - a noticeable narrowing of the penis, like an hourglass or band around the penis, or a folding, like a hinge, of the penis during sexual intercourse.

In addition, respondents with chordee (congenital curvature of the penis) were eligible only if they reported (1) one of the above-mentioned symptoms other than a significant bend or curve of the penis or (2) a diagnosis of PD. A total of 27 patients had chordee and 12 of them were eligible for Phase 2, and all of them met additional criteria for inclusion in the study. Specifically, 2 of them reported diagnosis of PD, 1 had received treatment for PD, and 9 had other qualifying symptoms of PD.

### 2.3. Sampling Method for the Phase 2 Peyronie's Disease Survey

Participants invited to complete Phase 2 were selected using a stratified random sampling approach based on the following 2 strata: (1) participants reporting treatment and/or diagnosis (Stratum 1) and (2) participants reporting symptoms only (Stratum 2). To ensure an adequate sample size to evaluate treatment patterns, respondents reporting prior treatment or a diagnosis for PD in Phase 1 were oversampled in Phase 2. 

After excluding respondents who were no longer active in the KN panel, all respondents from Stratum 1 (diagnosis and/or treatment) were invited to complete the Phase 2 PD survey. In addition, to ensure that at least 200 completed patient surveys could be obtained, 314 participants from Stratum 2 (symptoms only) were randomly selected to complete the Phase 2 PD survey.

### 2.4. Questionnaire Content

The Peyronie's portion of the screener administered in Phase 1 contained questions assessing the presence of various penile-related symptoms, diagnosis of PD, and past surgical or nonsurgical treatments for penile-related symptoms (see the Appendix).

The Peyronie's survey in Phase 2 focused on the presence and severity of current symptoms, family history, and treatment history for penile symptoms (e.g., specific symptom first noticed, time from noticing first symptom until seeking medical treatment, other symptoms noticed, types of treatment received and success of each treatment type, length of treatment/duration of care, number of surgeries and/or nonsurgical procedures, diagnoses received, and types of physicians treating and/or diagnosing the disease).

### 2.5. Data Analysis

#### 2.5.1. Prevalence Estimates

The prevalence estimates of PD were based on self-reported data from Phase 1 and adjusted to represent the US population by using statistical weights provided by KN. These weights are designed to (1) account for the known sources of deviation from an equal-probability selection design during formation of the KN panel, (2) reduce bias due to noncoverage of nontelephone households, and (3) reduce the nonresponse bias potentially introduced during data collection for outcomes highly correlated with demographic and geographic characteristics.

Three different prevalence estimates for PD were calculated. The numerators for the prevalence estimates of PD were derived using the following 3 criteria, ordered from most to least stringent, and the denominator was the number of individuals who completed Phase  1.

**Criterion 1**

Participant has received a diagnosis of PD from a doctor or other health care professional.

**Criterion 2**

Participant meets Criterion 1 or has ever had surgery and/or a nonsurgical procedure to correct the shape of the penis.

**Criterion 3**

Participant meets Criterion 2 or has penile plaque or a deformity as indicated by an affirmative response to at least 1 of the following: a lump or bump under the skin of the penis when penis is not erect, unusual firmness or hardened tissue under the skin of the penis when penis is not erect, bend or curve when the penis is erect, indentation when the penis is erect, noticeable narrowing, like an hourglass, when the penis is erect, or folding like a hinge when the penis is erect

A participant with chordee did not meet PD criteria unless he also reported at least 1 of the following symptoms: lump, firmness, indentation, narrowing or folding, or a diagnosis of PD by a physician or other health care provider.

#### 2.5.2. Analysis of Phase  2 Peyronie's Disease Survey

As described previously, the PD survey was administered in Phase  2 to a stratified random sample of patients indicating a diagnosis of PD, and/or treatment for penile symptoms (Stratum 1), or presence of penile symptoms (Stratum 2). Because patients reporting a diagnosis or treatment were oversampled, summary statistics (i.e., means or percentages) were calculated using weighted averages of the stratum-specific estimates to obtain an overall estimate among the eligible participants across the 2 strata (treatment/diagnosis and symptom only) [30]. No imputations were made for missing values. When calculating percentages, participants who did not answer a particular question were excluded from the denominator for that question. 

## 3. Results

Of the 16,000 men aged 18 years and older who were randomly selected from the pool of KN panel members and invited to participate in Phase  1, a total of 11,420 completed the screening survey for a response rate of 71%. In Phase  1 of the study, 415 PD patients were identified (101 of them reported a PD diagnosis and/or surgical/nonsurgical treatment) and invited to complete a full survey in Phase  2. Of those invited to participate, 283 (68%) consented and completed the PD full survey in Phase  2.

### 3.1. Demographic Characteristics

Table 2 presents unweighted demographic characteristics for all respondents in Phase  1 as well as respondents to the PD survey in Phase  2. The mean age of all respondents in Phase  1 was 52.7 (SD = 15.0) years. In Phase  2, the mean age of the respondents who had received a diagnosis or treatment was 59.6 (SD = 11.9) years, while the mean age for respondents who had symptoms but were not diagnosed/treated was 49.6 (SD = 17.0) years. The majority of respondents across both phases were white (86%) and had completed at least a high school level of education. Respondents with symptoms but no diagnosis/treatment tended to be younger, Black and Hispanic, and less likely to have post-high-school education compared to those with diagnosis or treatment.

#### 3.1.1. Prevalence Estimate

The estimated prevalence of PD based on the 3 criteria ranged from 0.5% to 13%, ordered from most to least stringent criteria (Table 3).

### 3.2. Peyronie's Disease Symptoms and Treatment Patterns

Results from the Phase  2 survey indicate that 21% of patients with PD diagnosis, treatment, or symptoms reported having a lump or bump under the skin of their penis, and 21% reported unusual firmness or hardened tissue under the skin of their penis (Table 4). Thirty-seven percent indicated that these symptoms affected the shape of their penis. Most patients (95%) reported no painful erections or pain during intercourse. However, 36% reported penile-related symptoms that interfered with sexual activities. The most common first penile symptom noticed was a significant bend or curve (32%). Other first penile-related symptoms commonly reported include erections not hard enough for sexual intercourse (20%), head of penis less hard (12%), lump or bump under the skin (11%), or shortening of penis (10%). 

Of the 283 PD patients, 128 reported seeing a doctor for the treatment of penile-related symptoms at some time during their lifetime. Among these patients, 68% reported seeing a doctor when they first noticed symptoms; the mean time to see the doctor was 16.8 (SD = 5.5) months. One-third (32%) of patients spoke with a doctor at a later time; the mean time to see the doctor was 46.2 (SD = 8.4) months. The majority (73%) of PD patients seeking treatment first saw a primary care physician (PCP), and 22% first saw a urologist/urology surgeon (note: from here forward, the term urologist will include urology surgeon). The most common symptoms that prompted seeking treatment were erections not hard enough for intercourse (51%), lump or bump under the skin of the penis (13%), and significant bend or curve in the penis (10%). 

Only 8% of treatment-seeking patients reported receiving a diagnosis of PD from the first doctor seen for penile symptoms; 48% reported receiving a diagnosis of ED. Among the few that were diagnosed with PD, 59% received the diagnosis from a urologist, and 41% received the diagnosis from their PCP. 

Treatment was often not given (49%), and patients were often advised to “wait and see” (25%) from the first doctor seen for penile symptoms. When treatment was eventually recommended at any doctor visit, the most commonly reported treatments were ED treatment (17% of those who saw a doctor), topical gel applied to the skin of the penis (7%), vitamin B or potaba (7%), vacuum or stretching therapy (5%), and vitamin E (5%). The mean time to treatment from the first noticed symptom was 8.9 (SD = 6.7) months (topical gel) to 37.0 (SD = 12.3) months (ED therapy).

#### 3.2.1. Summary of Surgical Procedures

Sixteen PD patients reported undergoing surgery for PD; of these, 14 patients reported 1 surgery and 2 patients reported 3 or more surgeries. A urologist performed the initial surgery in 81% (13/16) of participants. The mean time from first noticing a penile symptom until the first surgery was 31.8 months (SD = 64.2 months). The mean time from first diagnosis to first surgery was 6.3 months (SD = 6.8 months). Among respondents, 5 patients reported receiving a prosthetic penile implant, and 2 patients reported surgical grafting (6 patients reported “other” surgical procedure). Of the 16 patients who had surgery performed, 13 reported that the first surgery corrected the penile-related symptoms, and 11 reported that symptoms did not return, and no new symptoms were reported following surgery.

#### 3.2.2. Summary of Nonsurgical Injection Procedures

Twelve PD patients reported injections to treat penile symptoms; 10 patients reported 1 series of injections, and 2 patients reported 5 or more injection series. The first injection series was performed by a urologist in 42% (5/12) of participants, a PCP in 25% (3/12) of patients, and a sexual medicine specialist in 25% (3/12) of patients. Among 10 respondents who provided information around timing of injections, the first injection procedure was an average of 9.7 months (SD = 14.4 months) from the time the first symptom was noticed. Three of these 10 respondents were diagnosed, and the average time to the first injection series from the time of first diagnosis was 2.3 months (SD = 2.5 months). Four respondents knew the type of medication received in the first injection series; 2 reported verapamil, 1 reported testosterone, and 1 reported an unspecified “other” injection. Six out of 12 patients who had injections to treat penile symptoms reported that the injections corrected the penile-related symptoms, and 4 patients reported that symptoms did not return, and no new symptoms were reported following the procedure.

## 4. Discussion

Although PD has been recognized for over 200 years, no consensus exists with regard to the etiology, prevalence, treatment, or even the definition of this condition. The current study, the first large-scale, web-based population survey of PD among men in the US, showed the estimated prevalence of PD in the US to range from 0.5% (diagnosis of PD) to 13% (diagnosis, treatment, or penile symptoms) using criteria described previously. The estimate of 0.5% was based on respondent-reported diagnosis by a physician. The study did not collect information on what exams were used by the physician to arrive at the diagnosis. However, this number may be underestimated because most of the responders of the study did not see a physician even though they experienced penile symptoms. When including those reporting penile symptoms, the prevalence increased to 13%. The higher prevalence of symptoms of PD compared with the actual diagnosis suggests that PD may be underdiagnosed in the US. Additionally, diagnosis of ED and high usage of ED-related treatments may actually indicate misdiagnosis of PD symptoms. Underreporting of PD may occur because affected individuals are not comfortable discussing symptoms with health care professionals or are unaware of effective treatment options. In addition, health care professionals may not recognize symptoms when presented by patients, or they may not perform physical exams themselves and may instead refer patients to a specialist. Not all health care professionals may be properly trained in this area of research, and thus, they may be unaware of available treatments. 

It has recently been shown that PCPs and urologists may not be aware of recent findings related to the prevalence of PD, its association with ED, and its responsiveness to available treatments [31]. Results of a survey by LaRochelle and Levine found that, contrary to new information regarding PD, many physicians (63% of PCPs and 41% of urologists) continue to believe the prevalence of PD is below 1%, and 17% of PCPs and 38% of urologists believed that the disease remits spontaneously in more than 50% of cases [31], despite the lack of data from available research supporting these beliefs.

Among PD patients who reported a diagnosis, treatment, or penile symptoms related to PD, interference in sexual activities was reported by approximately one-third. Thirty-one percent of patients reported either a lump/bump or an unusual firmness or hardened tissue under the skin of the penis. While this number of patients who identified a palpable plaque may seem low, a study of more than 1,500 patients with PD found similar number of patients reporting plaques (39%), so these results may be more common than originally suspected [32]. Penile pain is an important consideration in patients with PD, particularly in the early, acute phase of PD [33, 34]; however, pain was not declared by many patients in this study. We cannot make definitive conclusions about the absence of reported pain because this study asked about current symptoms and symptoms first noticed, not the symptoms *ever* experienced. It is possible that some patients experienced pain at some point during the course of the disease, but this study was not intended to capture all symptoms ever experienced.

Regardless of the fact that patients reported a wide array of penile symptoms, fewer than half of PD patients reported seeking treatment. Many patients may be reluctant to come to their physician for treatment and diagnosis of this embarrassing condition [25]. Several factors have been identified that predict which patients are more inclined to delay treatment, including older age, being in a long-term relationship, having a partner, being heterosexual, and the presence of simple penile deformity [35]. Of those seeking medical treatment for penile symptoms, most initially saw a PCP, and the average duration of time to a doctor visit was within a year and a half of noticing a penile symptom. Approximately one-third of PD patients did not see a doctor until 4 years after the emergence of penile symptoms. Consistent with the possibility of underdiagnosis and undertreatment of PD in the US, only 8% of PD patients received a diagnosis of PD from the first doctor seen; the most frequent diagnosis was ED. Approximately three-quarters of the patients received no treatment from the first doctor seen, and 15% received treatment related to ED. These results further indicate that PD is not well understood, and the time to appropriate diagnosis and treatment can be long.

The observed treatment patterns for patients with PD include surgical and nonsurgical procedures. There are currently no FDA-approved nonsurgical options. Therefore, physicians have to base treatment recommendations for nonsurgical options on limited placebo-controlled clinical trial support [9, 36, 37]. This may lead some physicians to offer expectant management as opposed to potentially ineffective nonsurgical therapies [38]. Penile operations and injection treatments were uncommon among PD patients. Urologists most frequently performed these procedures, followed by PCPs and sexual medicine specialists for injection procedures. The median duration of time to receive treatment following diagnosis of PD was approximately 2 months earlier for injection procedures compared with surgery and, from noticing the first symptom, was approximately 2 and a half months earlier for injection procedures compared with surgery. Though surgery may be effective for many patients, it may be associated with complications and the possibility of penile shortening with some procedures [14–16]. It is generally reserved for patients in the chronic phase of PD with deformity and interference in sexual function, and so effective nonsurgical treatment for PD is needed [14].

A major strength of the current study is the use of a web-based survey to recruit a large, population-based sample of participants who were representative of the general US adult male population, which has been lacking in the current PD epidemiological literature. The survey had a high response rate, and the participant responses provided the first available population-based prevalence and incidence estimates for PD in the US. A weakness of the current study is the small number of participant responses for some of the measured outcomes (e.g., surgery). Oversampling of more severe cases of PD may have occurred in the Phase  2 survey; all survey respondents in Phase  1 who reported a diagnosis of PD or treatment for PD were invited to participate in Phase  2, whereas a subsample of participants reporting only symptoms were randomly selected for participation in Phase  2. Finally, participants self-reported their diagnoses and symptoms of PD through a process that allowed for respondent confidentiality (respondents were able to skip over any questions they did not feel comfortable answering or stop the survey at any time) which may have led to under- or overreporting on the survey. Additional problems with patient self-reporting could include misinterpretation of PD-specific symptoms or imperfect recall when symptoms occurred.

## 5. Conclusion

This study is the first large-scale, web-based population survey of PD among a representative sample of US adult men. The patient-reported prevalence of PD ranged from 0.5% to 13%, depending on how the occurrence of PD was defined. One-third of participants reporting PD also reported interference with sexual activities. More than 90% of PD patients who sought medical care for penile symptoms did not receive a diagnosis of PD, and approximately 75% did not receive any treatment from the first doctor seen. Study findings suggest that PD may be underdiagnosed and undertreated in the US and point to the need for better awareness of PD and related treatment options among health care professionals.

## Supplementary Material

Based on responses to particular items, not all respondents were asked all questions, according to skip patterns. Additionally, some of the questions may have looped back based on number of symptoms/doctors reported, etc.

## Figures and Tables

**Table 1:** Prevalence of Peyronie's disease from the literature.

	Patient population	Prevalence (%)
Arafa et al. 2007 [17]	Diabetic patients with erectile dysfunction	20.3
Mulhall et al. 2004 [21]	Men screened for prostate cancer	8.9
El-Sakka 2006 [18]	Patients with erectile dysfunction	7.9
La Pera et al. 2001 [19]	General population	7.1
Rhoden et al. 2001 [22]	Men older than 50 years undergoing prostate cancer screening	3.67
Schwarzer et al. 2001 [23], Sommer et al. 2002 [24]	General population	3.2
Lindsay et al. 1991 [20]	General population	0.39

**Table 2:** Demographic characteristics of Phases  1 and 2 male participants.

			Phase 2 participants (*n* = 283)	
Characteristic	Category	Phase 1 participants (*n* = 11,420)	With diagnosis/treatment (*n* = 84)	With symptoms and no diagnosis/treatment (*n* = 199)
		*n* (%)	*n* (%)	*n* (%)

Age (years)	Mean (SD)	52.7 (15.0)	59.6 (11.9)	49.6 (17.0)
	Median	53	60	50
	Range	18–101	20–87	18–89

Race	White, non-Hispanic	9803 (86%)	76 (90%)	137 (69%)
	Black, non-Hispanic	571 (5%)	3 (4%)	25 (13%)
	Other, non-Hispanic	544 (5%)	5 (6%)	14 (7%)
	Hispanic	502 (4%)	0 (0%)	23 (12%)

Education	Less than high school	588 (5%)	3 (4%)	16 (8%)
	High school	2098 (18%)	8 (10%)	57 (29%)
	Some college	3898 (34%)	32 (38%)	58 (29%)
	Bachelor's degree or higher	4836 (42%)	41 (49%)	68 (34%)

SD: standard deviation.

**Table 3:** Estimated prevalence of Peyronie's disease in US males in 2007.

	Phase 1 respondents (*n* = 11,420)	
Disease criteria	Prevalence (%)	95% CI
Criterion 1: Diagnosis of PD	0.5	0.4–0.7
Criterion 2: Diagnosis or treatment of PD	0.8	0.5–1.0
Criterion 3: Diagnosis, treatment, or any symptom of PD	13.1	12.0–14.1

CI: confidence interval.

Note: Prevalence estimates are weighted estimates from 11,420 male respondents.

**Table 4:** Summary of Peyronie's disease symptoms in participants with symptoms, treatment, or diagnosis (*n* = 283).

	Statistic or category	Participants (*n* = 283) (*n*, %*)
Lump or bump under skin of penis	Yes	60 (21)
	No	222 (79)
	Missing	1

Unusual firmness or hardened tissue under skin of penis	Yes	69 (21)
	No	210 (79)
	Missing	4

Lump/bump, firmness, or hardened tissue affect the shape of penis (if yes to either 1 of the above 2 symptoms, *n* = 87)	Yes	48 (37)
	No	39 (63)

Significant bend or curve compared to a younger age (erect penis)	Yes	177 (59)
	No	106 (41)

First penis-related symptom noticed	Lump or bump under the skin	24 (11)
	Area of unusual firmness or hardened tissue under the skin	13 (4)
	Significant bend or curve not previously noticed	95 (32)
	Indentation on 1 or both sides	7 (2)
	Noticeable narrowing of the penis (hourglass or bandlike)	4 (2)
	Hinge-like folding of penis during sexual intercourse	7 (3)
	Shortening of penis	20 (10)
	Head of penis less hard	26 (12)
	Painful erections	4 (<1)
	Pain during sexual intercourse	5 (2)
	Erections not hard enough for sexual intercourse	51 (20)
	Other	8 (3)
	Missing	19

*Percentages are weighted averages of the stratum-specific estimates.

## Appendix

### A. Peyronie's Disease Survey

For more details see Supplementary Material available online at doi:10.1155/2011/282503.
