# Supplementary material for: A Population-Based Study of Peyronie's Disease: Prevalence and Treatment Patterns in the United States
Source: Adv Urol. 2011 Oct 23;2011:282503. doi: 10.1155/2011/282503 (PMC3202120; doi:10.1155/2011/282503)
Supplement: Supplementary file 1 — Based on responses to particular items, not all respondents were asked all questions, according to skip patterns. Additionally, some of the questions may have looped back based on number of symptoms/doctors reported, etc. [file 282503.f1.pdf]

Appendix: Peyronie's Disease Survey

Based on responses to particular items, not all respondents were asked all questions, according to skip patterns. Additionally, some of the questions may have looped back based on number of symptoms/doctors reported, etc.

SCREENING QUESTIONS		***RESPONSES***
**Asked on Phase I survey (screener) and confirmed in Phase II survey**	**Lump or bump under the skin of non-erect penis (not including genital warts, pimples, or blisters)**	Yes, No
	**Unusual firmness or hardened tissue under the skin of non-erect penis (not including genital warts, pimples, or blisters)**	Yes, No
	**Lump/bump, firmness, or hardened tissue affect the shape of erect penis**	Yes, No
	**Development of a new significant bend or curve of erect penis**	Yes, No
	**Describe change in erect penis compared to when younger**	☐ An indentation has developed on one or both sides of penis<br>☐ Noticeable narrowing of penis<br>☐ Penis folds during sexual intercourse<br>☐ Penis is shorter than it used to be.<br>☐ The head of penis is less hard than it used to be<br>☐ None of the above
**Treatment (surgery or injections) to correct the shape of penis**		Yes, No
**Diagnosis of Peyronie's disease**		Yes, No
**Diagnosed of chordee or congenital curvature of the penis**		Yes, No
DIAGNOSIS OF PEYRONIE'S QUESTIONS		
**Ever been told by a doctor or other healthcare professional that you have Peyronie's disease**		Yes, No
**Time of first diagnosis of Peyronie's disease**		Month/year
**Doctor or healthcare professional who made diagnosis of Peyronie's disease**		☐ Primary Care Physician (PCP)<br>☐ Urologist or Urology Surgeon<br>☐ Plastic Surgeon<br>☐ Sexual Medicine or Sexual Health Specialist<br>☐ Dermatologist<br>☐ Other, specify:<br>☐ Not sure
SYMPTOM QUESTIONS		
**Experience painful erections**		Yes, No
**Experience pain during intercourse**		Yes, No
**Difficulty achieving an erection hard enough for intercourse**		Yes, No
**Penis-related symptoms interfere with sexual activities**		Yes, No
Questions About First Symptom Noticed and First Doctor Seen **(**If more than one doctor/healthcare provider was seen, questions were repeated for each doctor/healthcare provide seen)		
**First penis-related symptom noticed**		☐ A lump or bump on penis<br>☐ Hardened tissue on penis<br>☐ A significant bend/curve in penis<br>☐ An indentation in penis

	☐ An narrowing or hourglass shape of penis<br>☐ Folding of penis during sex<br>☐ Penis shorter than it used to be<br>☐ The head of penis being less hard than it used to be<br>☐ Painful erections<br>☐ Pain during sexual intercourse<br>☐ Erections not hard enough for sexual intercourse<br>☐ Other, specify:
**Date when first was noticed symptom**	Month/year
**Consultation with a doctor/healthcare professional when symptom <u>first</u> noticed**	Yes, No
**Type of doctor/healthcare professional seen**	☐ PCP<br>☐ Urologist or Urology Surgeon<br>☐ Plastic Surgeon<br>☐ Sexual Medicine or Sexual Health Specialist<br>☐ Dermatologist<br>☐ Other, specify:
**Date of consultation with first doctor**	Month/year
**<u>Other</u> symptoms discussed with doctor**	☐ A lump or bump on penis<br>☐ Hardened tissue on penis<br>☐ A significant bend/curve in penis<br>☐ An indentation in penis<br>☐ An narrowing or hourglass shape of penis<br>☐ Folding of penis during sex<br>☐ Penis shorter than it used to be<br>☐ The head of penis being less hard than it used to be<br>☐ Painful erections<br>☐ Pain during sexual intercourse<br>☐ Erections not hard enough for sexual intercourse<br>☐ Other, specify:
**Diagnosis given for penis-related symptoms**	☐ Chordee or congenital curvature of the penis<br>☐ Erectile Dysfunction<br>☐ Peyronie's disease<br>☐ Other, specify:<br>☐ None of the above
**Date of diagnosis of Peyronie's disease (if applicable)**	Month/year
**Types of treatment received from doctor**	☐ Acupuncture<br>☐ Colchicine<br>☐ Electric current therapy<br>☐ Radiation<br>☐ Shots or injections into the penis<br>☐ Surgery<br>☐ Topical gel<br>☐ Ultrasound or laser therapy

	☐ Vacuum or stretching therapy<br>☐ Vitamin B or Potaba<br>☐ Vitamin E<br>☐ Wait and see<br>☐ Other, specify:<br>☐ No treatment
**Extent to which the symptom(s) changed with treatment**	☐ Got better<br>☐ Got worse<br>☐ Stayed the same<br>☐ Initially got better then got worse again
**Improvement of symptom without treatment**	Yes, No
**Still seeing doctor for penis-related symptoms**	Yes, No
**Duration of care for penis-related symptoms**	Months/years
TREATMENT-RELATED QUESTIONS<br>(Questions were only asked if applicable based on responses to previous treatment-related items)	
**Number of surgeries to treat penis-related symptoms**	☐ Never<br>☐ 1 time<br>☐ 2 times<br>☐ 3 times<br>☐ 4 times<br>☐ 5 or more times
**Type of doctor/healthcare professional who performed surgery**	☐ Urologist or Urology Surgeon<br>☐ Plastic Surgeon<br>☐ Other, specify:<br>☐ Not sure
**Date of surgery for penis-related symptoms**	Month/year
**Type of surgery to treat penis-related symptoms**	☐ Plication surgery or Nesbit procedure<br>☐ Surgical grafting<br>☐ Prosthetic Penile Implant<br>☐ Other, specify:<br>☐ Don't know<br>☐ None of the above
**Correction of problem**	Yes, No
**Response to surgery**	☐ The symptoms came back<br>☐ New symptoms appeared<br>☐ The symptoms have not come back and no new symptoms have appeared
**Time of recurrence/new symptoms after surgery**	Months/years after surgery
**Number of <u>series</u> of shots or injections to treat your penis-related symptoms**	☐ None<br>☐ 1 series<br>☐ 2 series<br>☐ 3 series<br>☐ 4 series<br>☐ 5 or more series
**Type of doctor/healthcare professional that administered each series**	☐ Primary Care Physician (PCP)<br>☐ Urologist or Urology Surgeon<br>☐ Plastic Surgeon<br>☐ Sexual Medicine or Sexual Health

	Specialist<br>☐ Dermatologist<br>☐ Other, specify:<br>☐ Not sure
**Date of series of shots or injections to treat the symptom(s) in your penis**	Month/year
**Medication received in series of shots or injections**	☐ Collagenase<br>☐ Corticosteroids<br>☐ Interferon<br>☐ Testosterone<br>☐ Verapamil<br>☐ Vitamin B or Potaba<br>☐ Other, specify:<br>☐ Not sure<br>☐ None of the above
**Number of shots with the series**	☐ 1 injection<br>☐ 2-6 injections<br>☐ 7-12 injections<br>☐ 13+ injections<br>☐ Not sure
**Correction of problem**	Yes, No
**Response to shots**	☐ The symptoms came back<br>☐ New symptoms appeared<br>☐ The symptoms have not come back and no new symptoms have appeared
**Time of recurrence/new symptoms after last shot or injection**	Months/years after last shot of series
